# Trough concentrations of cabotegravir and rilpivirine and their association with detectable viral load in people with HIV on long-acting treatment

**DOI:** 10.1007/s15010-025-02577-x

**Published:** 2025-07-04

**Authors:** Sebastian Noe, Ulrich Seybold, Farhad Schabaz, Ariane von Krosigk, Carmen Wiese, Eva Wolf, Celia Jonsson-Oldenbüttel, Anna Ivanova

**Affiliations:** 1 MVZ München am Goetheplatz, Munich, Germany; 2 Department of Medicine IV, University Hospital, LMU Munich, Germany; 3 Institute for Data Science, UHasselt, Hasselt, Belgium

## Abstract

**Background:**

Cabotegravir (CAB) and rilpivirine (RPV) constitute the first complete non-oral ART regimen for HIV-1 treatment. Due to virologic failure (VF) with resistance in clinical trials, concerns persist regarding broader use in clinical practice. In particular, the role of trough drug concentrations in relation to viremia and VF remains unclear. This study explored the association between CAB and RPV trough concentrations in a retrospective, single-center study.

**Methods:**

We retrospectively analyzed data from the HIV research and clinical care center MVZ München am Goetheplatz, Germany. Inclusion criteria were CAB and RPV long-acting therapy every 8 weeks without additional ART and availability of drug concentrations within 7 days before the next administration. A modified Wilcoxon test assessed differences in concentrations between samples with HIV-1 RNA < 20 vs. ≥20 copies/mL. Odds ratios (ORs) were estimated using generalized estimation equation (GEE) models, and ROC analysis identified potential alternative drug concentration thresholds.

**Findings:**

A total of 737 samples from 185 individuals were included. Median CAB concentrations were 1,480 µg/L (IQR: 1,097–1,955) vs. 1,180 µg/L (879–1,570) for samples with HIV-1 RNA levels < 20 copies/mL vs. ≥ 20 copies/mL, respectively (*p* = 0.001); for RPV, 77 µg/L (53–107) vs. 63 µg/L (47–87) (*p* = 0.001). Using ROC-derived thresholds, low concentrations of CAB (< 1,240 µg/L) or RPV (< 76 µg/L) were found in 11.5% and 25.4% of samples, respectively, and associated with ORs of 2.4 (1.5–4.0) and 2.3 (1.4–3.8) for HIV-1 RNA ≥ 20 copies/mL.

**Interpretation:**

Lower CAB and RPV concentrations were associated with viremia, particularly using the ROC-derived thresholds. Among individuals with VF and available drug concentration data, 87.5% had at least one drug below these thresholds. Further research on therapeutic drug monitoring is warranted.

## Background

The second-generation integrase inhibitor (INI) cabotegravir (CAB) together with the non-nucleoside reverse transcriptase inhibitor (NNRTI) rilpivirine (RPV) has become the first complete non-oral antiretroviral treatment (ART) regimen approved for HIV-1 therapy. However, unlike in clinical trials with other second-generation INIs, the occurrence of virologic failure (VF) with resistance was observed [1, 2], raising concerns about its broader use in clinical routine. Based on the observations in these trials, some risk factors have been identified that were associated with a higher risk of VF, including baseline resistance-associated mutations (RAMs) for RPV, subtype A6, and a BMI > 30 kg/m² [3].

While applying the aforementioned criteria in patient selection seems to have led to a decrease in the VF rates (indicated by the lower rates of VF in more recent studies, where RAMs and subtype were considered in the inclusion and exclusion criteria), there are still some people experiencing virologic failure in the absence of any of these. This raises the question of additional potential risk factors. Particular interest still continues to revolve around the role of (trough) concentrations of both drugs and their association with viremia and VF, as results published so far remain inconclusive: while some authors have reported associations of drug (trough) concentrations and the occurrence of viremic events [4, 5], others could not verify these findings [6] and a recent expansion of the aforementioned multivariate analysis questioned the predictive value of trough levels for VF in people on CAB & RPV [7]. Therefore, more data is needed to understand the meaning of therapeutic drug monitoring (TDM) in CAB & RPV.

This study aimed to explore the association between the trough concentrations of CAB and RPV measured in clinical routine in a retrospective, single-center study. The primary outcome was the comparison of trough concentrations of CAB & RPV in samples with and without detectable viral load, assuming that concentrations will be lower in samples with detectable viral load. The secondary endpoint was the comparison of the number of samples with and without detectable viral load between those with low versus normal or high concentrations of CAB and/or RPV. Exploratory endpoints included the derivation of alternative cut-offs for CAB- and RPV concentrations and the repetition of the secondary outcome analyses based on these new thresholds.

## Methods

### Design and study sample

This retrospective study utilized routinely collected data from a single HIV-research and clinical care center (MVZ München am Goetheplatz) in Munich, Germany. Data were extracted from electronic patient files. Inclusion criteria were treatment with CAB & RPV long-acting every 8 weeks without any additional antiretroviral agents in people living with HIV-1 and availability of CAB and RPV concentrations measured within a maximum of 7 days before the next administration. Exclusion criteria were missing data for any of the two concentrations, or HIV-1 RNA measurement. HIV-1 RNA was measured using the Cobas 6800 system (Roche Diagnostics, Mannheim, Germany), while concentrations of CAB and RPV were determined using liquid chromatography-tandem mass spectrometry. Multiple measurements per patient were allowed over time.

### Methodology and statistics

For descriptive statistics, medians with the 25th and 75th percentile (IQR), and absolute frequencies and percentages (%) were used for continuous and categorical variables, respectively.

For the primary outcome, an adapted Wilcoxon test for clustered data (introduced by allowing for multiple measurements per person) as suggested by Rosner, Glynn, and Lee [8] was used under a one-sided hypothesis, assuming lower concentrations in the samples with HIV-1 RNA ≥ 20 copies/mL. Concentrations below 800 µg/L for CAB and 35 µg/L for RPV were classified as “low” according to the reference range provided by the laboratory that carried out the measurements by liquid chromatography - tandem mass spectrometry.

For the secondary outcome, number of samples with and without low concentrations for CAB & RPV were determined among samples with and without HIV-1 RNA ≥ 20 copies/mL, and odds ratios (OR) were calculated based on the coefficients of a generalized-estimation equation (GEE) model to account for repeated measurements.

For the exploratory outcome, receiver operating characteristics (ROC) were used to calculate sensitivity and specificity for each concentration of both drugs over the range of observed concentrations in terms of ‘predicting’ HIV-1 RNA (dichotomized into < 20 copies/mL and ≥ 20 copies/mL). ‘Optimal’ cut-off concentrations for CAB and RPV were determined by maximizing the sum of sensitivity and specificity.

Given the exploratory nature of this study, we set the significance level at 0.10 instead of the conventional 0.05 to reduce the risk of overlooking potentially relevant signals. This approach allows for a more liberal interpretation of statistical findings while acknowledging the increased likelihood of type I errors.

Statistical analyses were performed using R version 4.4.1 (The R Foundation for Statistical Computing, Vienna). The clusrank package was used for the non-parametric analyses of clustered data.

### General information

A large language model (ChatGPT 4o) was used for copy editing.

Parts of this work have been presented during the 2025 Conference on Retroviruses and Opportunistic Infections in San Francisco, California, USA.

This work did not receive any funding.

## Findings

### Exploratory data analysis and summary statistics

Overall, 737 samples from 185 people were included in the analysis. A summary of the key characteristics for the people included in the study and the samples analyzed can be found in Tables 1 and 2, respectively.

**Table 1:** Key characteristics of the people included in this study

Age at first injection [years], median (IQR)	45	(35.0; 52.0)
**missing**,** n (%)**	7	(3.8)
**Sex [ref.: female]**,** n (%)**	27	(15.2)
**missing**,** n (%)**	6	(3.3)
**Body mass index [kg/m²]**,** median (IQR)**	25.4	(23.4; 28.1)
**missing**,** n (%)**	48	(26.1)
**Ethnicity [ref.: Caucasian]**,** n (%)**	135	(77.6)
**missing**,** n (%)**	10	(5.4)
**Route of transmission [ref.: MSM]**,** n (%)**	122	(72.2)
**missing**,** n (%)**	15	(8.2)
**CD4 nadir [cells/µL]**,** median (IQR)**	327	(177.0; 467.5)
**missing**,** n (%)**	53	(28.8)
**History of AIDS defining condition [ref.: yes]**,** n (%)**	14	(8.4)
**missing**,** n (%)**	17	(9.2)
**Samples per person [n]**,** median (IQR)**	4	(3.0; 5.0)
**missing**,** n (%)**	0	(0.0)
**HIV-1 subtype [ref.: A6]**,** n (%)**	1	(0.7)
**HIV-1 subtype [ref.: B]**,** n (%)**	92	(62.2)
**missing**,** n (%)**	36	(19.6)
**Oral lead-in [ref.: yes]**,** n (%)**	120	(65.2)
**missing**,** n (%)**	0	(0.0)
**Time on CAB & RPV [weeks]**,** median (IQR)**	143.4	(82.6; 178.4)
**missing**,** n (%)**	1	(0.5)

CAB: cabotegravir; CD4: CD4-positive lymphocytes; IQR: interquartile range; MSM: men who have sex with men; ref: indicator of the category for which the results apply; RPV: rilpivirine

**Table 2:** Summary of the results for the 737 samples analyzed in this study

Cabotegravir trough concentration [µg/L], median (IQR)	1445	(1064.8; 1921.2)
**missing**,** n (%)**	0	(0.0)
**Rilpivirine trough concentration [µg/L]**, median (IQR)	75	(51.0; 105.0)
**missing**,** n (%)**	0	(0.0)
**CD4 [cells/µL]**, median (IQR)	756	(597.0; 968.0)
**missing**,** n (%)**	3	(0.4)
**HIV-1 RNA [ref.: ≥ 20 copies/mL]**, n (%)	87	(11.8)
**missing**,** n (%)**	0	(0.0)
**HIV-1 RNA [ref.: ≥ 50 copies/mL]**, n (%)	18	(2.4)
**missing**,** n (%)**	0	(0.0)

CD4: CD4-positive lymphocytes; IQR: interquartile range; ref: indicator of the category for which the results apply

Median concentrations (IQR) for people with HIV (PWH) with HIV-1 RNA < 20 copies/mL or ≥ 20 copies/mL were 1,480 µg/L (1,097; 1,955) or 1,180 µg/L (879; 1,570) for CAB (*p* = 0.001), and 77 µg/L (53; 107) or 63 µg/L (47; 87) for RPV (*p* = 0.001), respectively.

Median concentrations (IQR) for PWH with “target not detected” or “target detected” were 1,515 µg/L (1,130; 1,955) or 1,290 µg/L (939; 1,760) for CAB (*p* < 0.001), and 78 µg/L (54; 111) or 67 µg/L (48; 93) for RPV (*p* = 0.003), respectively. Association of drug concentrations with viremic episodes.

**Fig. 1:**
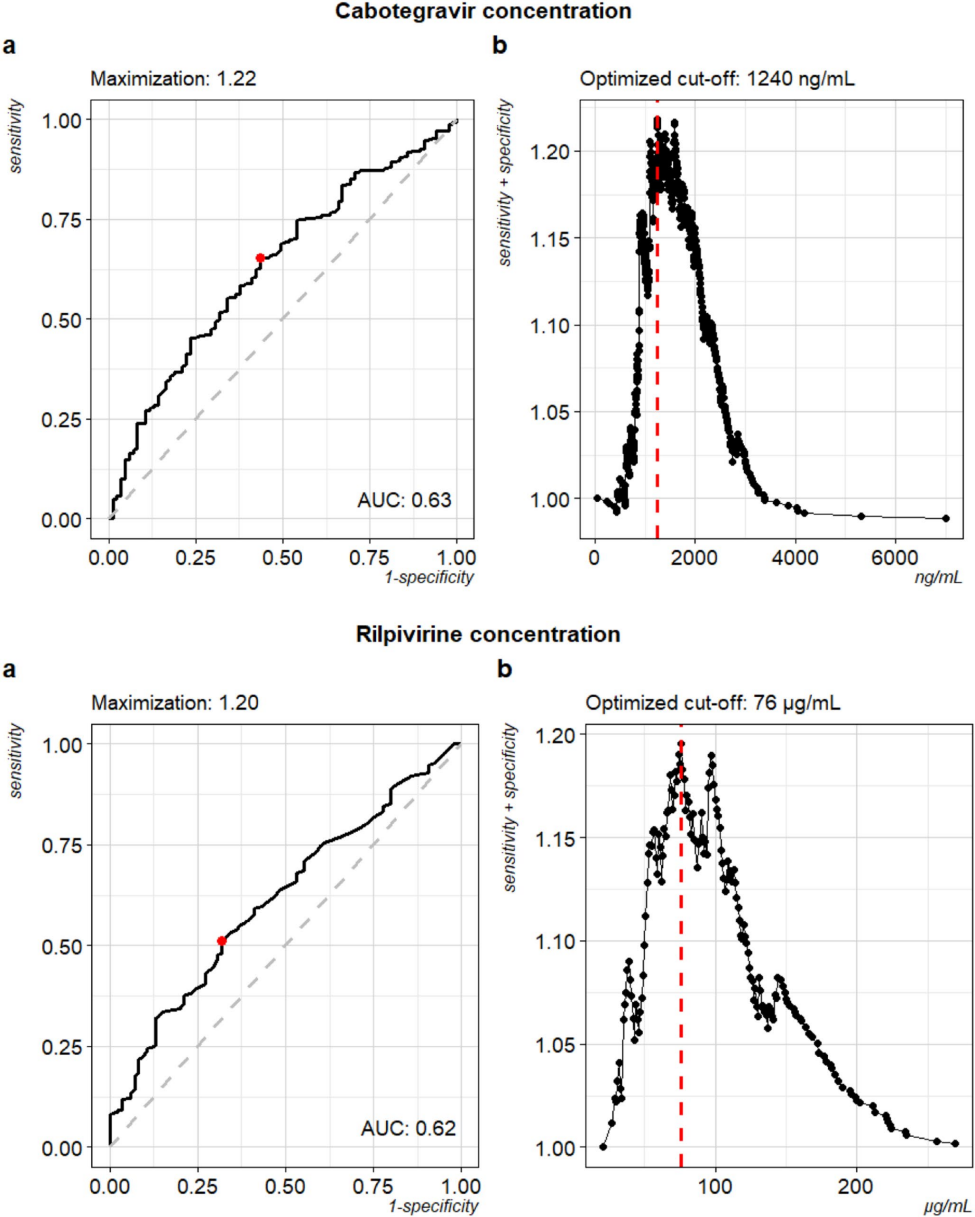
Receiver operating characteristics (ROC) for the sensitivity and 1-specificity throughout the range of possible ‘optimized’ thresholds for Cabotegravir (top) and Rilpivirine (bottom) on the left. The results of the maximized sum of sensitivity and specificity can be found on top of the figure. For both drugs, the right graphs depict the sums for sensitivity and specificity for a range of drug concentrations

Using 800 µg/L for CAB and 38 µg/L for RPV as thresholds to define low concentrations, 46 (6.2%) samples had low concentrations of CAB, 47 (6.4%) samples had low concentrations of RPV, and 17 (2.3%) had low concentrations for both drugs. 11/64 samples (17.5% [9.4; 29.5]) with low CAB concentrations and 74/677 samples (10.9% [8.7; 13.6]) with normal CAB concentrations had HIV-1 RNA ≥ 20 copies/mL, respectively. 12/64 samples (18.8% [10.5; 30.8]) with low RPV concentrations and 73/676 samples (10.8% [8.6; 13.4]) with normal RPV concentrations had HIV-1 RNA ≥ 20 copies/mL, respectively. Low concentrations for CAB and RPV were associated with odds ratios of 1.7 (0.8; 3.5) and 1.9 (0.9; 3.8) for HIV-1 RNA ≥ 20 copies/mL, respectively. Thresholds found by maximizing the sums of sensitivity and specificity were 1,240 µg/L and 76 µg/L for CAB and RPV (Fig. 1), respectively. Using these thresholds, 85 (11.5%) samples had low levels of CAB, 187 (25.4%) had low levels for RPV, and 190 (25.8%) had low levels for both. 48/275 samples (17.5% [13.3; 22.6]) with low CAB concentrations and 37/465 samples (8.0% [5.7; 10.9]) with normal CAB concentrations had an HIV-1 RNA ≥ 20 copies/mL, respectively. 58/377 samples (15.4% [12.0; 19.5]) with low RPV concentrations and 27/363 samples (7.4% [5.0; 10.8]) with normal RPV concentrations had a HIV-1 RNA ≥ 20 copies/mL, respectively. Low concentrations of CAB or RPV were associated with odds ratios of 2.4 (1.5; 4.0) and 2.3 (1.4; 3.8) for HIV-1 RNA ≥ 20 copies/mL, respectively.

**Fig. 2:**
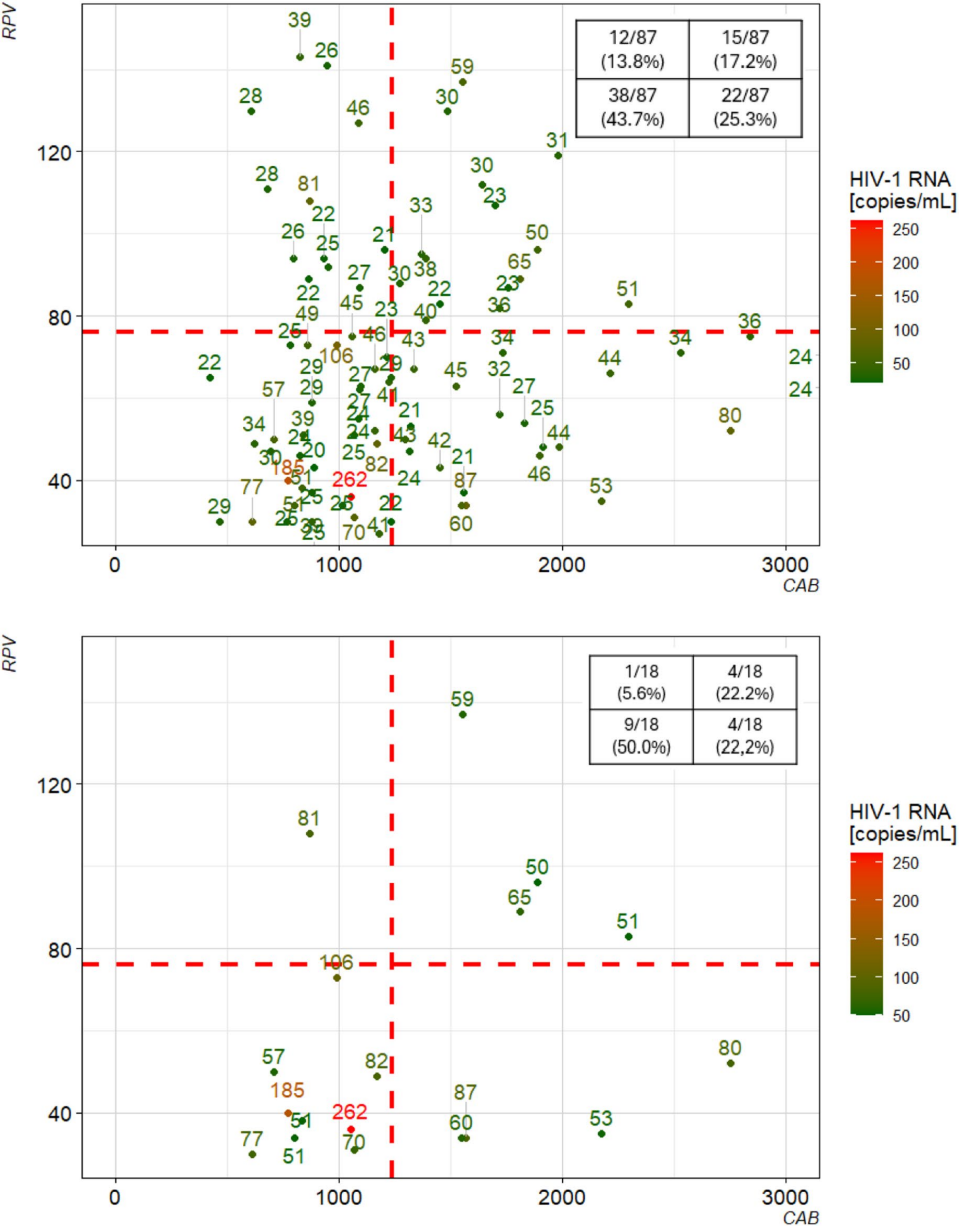
Scatter plot of measurement pairs for cabotegravir (CAB) and rilpivirine (RPV) concentrations for all samples with HIV-1 RNA ≥ 20 copies/mL (top) and ≥ 50 copies/mL (bottom). Red dashed lines indicate the data-derived thresholds for CAB and RPV, tables in the upper right corner of the plots indicate the number of samples per quadrant in relation to the number of samples with HIV-1 RNA ≥ 20 copies/mL (*n* = 87) or HIV-1 RNA ≥ 50 copies/mL (*n* = 18), respectively. Colors indicate the result of HIV-1 RNA quantification, also indicated by the numbers next to each measurement

Figure 2 depicts the division of all pairs of measurements with HIV-1 RNA ≥ 20 copies/mL into four quadrants, of which the left lower quadrant is the one for blood samples with low concentrations for both CAB and RPV.

### Interpretation

**Fig. 3:**
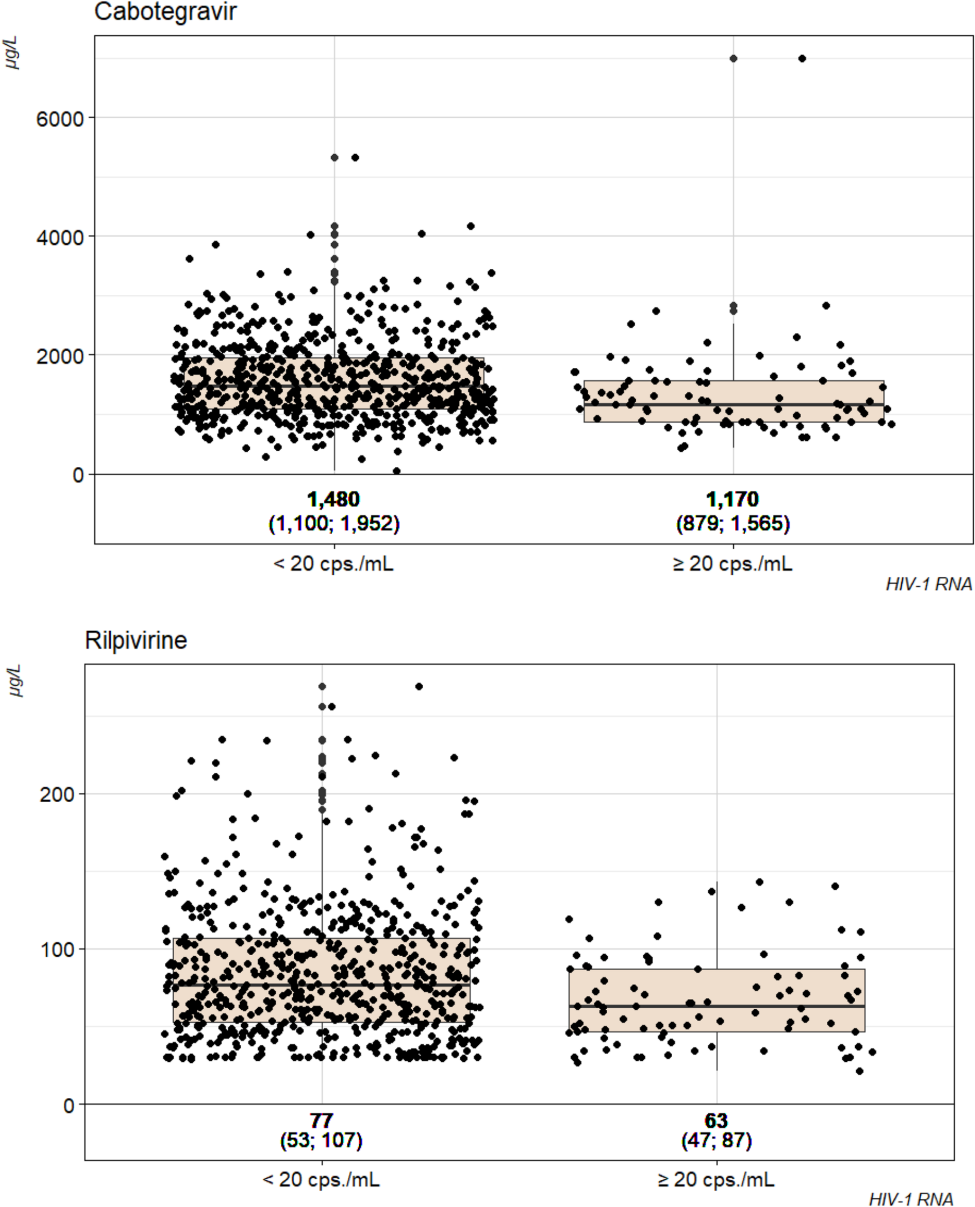
Box- and jitter-plots of the concentrations of Cabotegravir and Rilpivirine by status of virologic suppression. Numbers below the plots indicate the median together with the interquartile ranges for the respective drug

In our study we found an association between drug concentrations of CAB and RPV and HIV-1 RNA measured in the same sample. Samples with HIV-1 RNA ≥ 20 copies/mL had on average lower concentrations for both CAB and RPV (Fig. 3). Likewise, when applying the thresholds of 800 µg/L for CAB and 35 µg/L for RPV, a higher proportion of samples with low drug concentrations had HIV-1 RNA ≥ 20 copies/mL. From a cohort in Spain, similar data were reported, where in four people with HIV-1 RNA ≥ 50 copies/mL lower concentrations of both drugs were found [5].

It should be noted that the trough concentrations for CAB (1,445 µg/L [1,064.8; 1921.2]) and RPV 75 µg/L (51.0; 105.0) found in our study were at the lower end of the range reported in clinical trials, ranging from roughly 1,500 to 1,800 µg/L and 70–100 µg/L [9, 10, 11], respectively. At least one other real-world study also reported lower drug levels than in clinical trials [6].

Since the optimal thresholds for CAB and RPV in the context of therapeutic drug monitoring (TDM) remain a topic of debate, we explored alternative cut-offs by applying ROC analyses, selecting the concentration that maximized the sum of sensitivity and specificity for the binary outcome (HIV-1 RNA < 20 copies/mL: yes vs. no). This led to thresholds of 1,240 µg/L and 76 µg/L for CAB and RPV, respectively. While both values are considerably higher than the ones used before, they are still lower than the ones reported from four-weekly dosing [11]. It has been advocated in models for oral RPV before that often-used thresholds might be too low and concentrations of up to 100 µg/L were discussed, however, including people without prior treatment [4]. Interestingly, a threshold of 100 µg/L was comparable in terms of sensitivity and specificity to 76 µg/L in our ROC analysis, leading to an only slightly lower sum of the two test performance characteristics (Fig. 1). It is noteworthy that the AUCs of both ROC analyses are quite low; however, it must be kept in mind that the contribution of both drugs might be relevant and that therefore the AUC for each single drug might not be indicative of the discriminatory ability of the combined use.

In this context, it should be noted that a trend toward higher efficacy with four-weekly compared to eight-weekly dosing has been observed [3], and that people with HIV (PWH) who were stable on CAB & RPV Q4W experienced virologic failure (soon) after switching to Q8W [10]. Both phenomena are most easily explained by the differences in pharmacokinetics, as all other risk factors should contribute equally in both settings.

When visualizing paired CAB and RPV measurements together with the data-derived thresholds for samples with HIV-1 RNA ≥ 20 copies/mL (Fig. 2), most samples fell into groups with low levels of at least one of the drugs. The highest detected viral loads were all found in the quadrant with low concentrations of both drugs. This quadrant also contained the highest proportion of samples with HIV-1 RNA ≥ 20 copies/mL. In contrast, only about 20% of samples with HIV-1 RNA ≥ 20 copies/mL had sufficient concentrations of both CAB and RPV. However, this also suggests that trough concentrations alone may not be sufficient to predict viremic episodes in PWH, a finding that has been noted previously [5].

### Limitations

Our study has several limitations. First, it is a retrospective study, meaning data collection occurred post hoc. However, the study site had implemented therapeutic drug monitoring for all individuals receiving CAB and RPV, ensuring that the primary outcome measure was assessed as a standard during virtually all visits. Second, our results are only based on trough levels, that don’t reflect the entire treatment period between two injections. However, they might be seen as a (good) surrogate, reflecting a minimum concentration during this time. Third, it is often argued that the Roche assay has a high variability compared to other tests in the field and therefore the results have to be interpreted with care. However, the following consideration is noteworthy in the context of our study: a higher variability adds more “noise” to the data, which makes it more difficult to detect a true signal. Finding such a signal is therefore even more likely to be “true”. This is particularly true as in the suppressed setting the variability will (almost) always be directed towards “false-high” viral loads, meaning that more often a detectable viral load will be found where there might be none in another assay. It should therefore be expected that more “false-high” HIV-1 RNA levels are detected, which is in favor of the null hypothesis and should therefore increase the type 2 error, instead of the type 1 error. In the same context it must be mentioned that low-level viremic events are not comparable to virologic failure. Due to the rare occurrence of virologic failure (none in this study sample), we had to focus on viremic events. However, for this reason we tried to validate our findings by applying our findings to the reported VFs from clinical trials and real-world data and found high rates of insufficient drug-levels. This still does not prove causality. Finally, all data were derived from a single clinical site, which may impact the generalizability of our findings. This is particularly relevant for the alternative drug concentration thresholds, as they may have been “overfitted” to the available data. From a clinical perspective, it is noteworthy that no virologic failure occurred among the individuals included in this study. This raises the question of whether low CAB and RPV concentrations are merely associated with viral blips and low-level viremia or if they serve as true predictors or risk factors for virologic failure. Low-level viremic events are not consistently linked to eventual virologic failure. However, when applying our thresholds to PWH with virologic failure in clinical trials (ATLAS-2 M, SOLAR) [1, 12] and real-world cohorts [5, 6, 13, 14, 15, 16, 17, 18, 19] where trough levels at the time of VF were reported, we found that in 25/28 cases (89.3%) low concentrations were present. After removing people with subtype A6 or major NNRTI resistance at baseline, 17/19 subjects (89.5%) and 18/19 subjects (94.7%) were low in either concentration for the 76 µg/L and 100 µg/L thresholds for RPV, respectively. For comparison: the expected proportion based on our sample would be 62.6% (59.0-66.1; *p* = 0.021 for a one-sided test for proportions, data not shown) and 78.2% (75.0-81.1; *p* = 0.086 for a one-sided test for proportions, data not shown) for the lower and higher thresholds of RPV, respectively. 9/18 (50%) people experiencing virologic failure had low levels for CAB and RPV as compared to an expectation of 27.7% based on the findings in our study sample (*p* = 0.021 for a one-sided test for proportions, data not shown). Lastly, it is likely that other factors like the size of the reservoir or intercurrent infections amongst others, could contribute to the occurrence of viremic episodes which has not been considered in the current analysis.

## Conclusions

In summary, our results indicate an association between CAB and RPV concentrations and the occurrence of viremic episodes, particularly when both drug concentrations are low (e.g., CAB < 1,240 µg/L and RPV < 76 µg/L). However, since the thresholds identified in our study are higher than those commonly used, their clinical utility remains uncertain, as many individuals without viremic episodes may also fall below these thresholds. A larger dataset with a more diverse study population could help to better define clinically relevant thresholds. Of note, the proportion of people with low CAB and/or RPV concentrations among people experiencing virologic failure reported in the literature seems to be higher than expected based on our study sample.

While drug concentrations may be linked to virologic suppression, other factors could be equally or even more relevant. This underscores the need to assess the contribution of drug concentrations while adjusting for other relevant factors. This consideration becomes particularly important in light of ongoing plans for a four-monthly dosing regimen of CAB and RPV.

## Appendix

**Table:** 

Study or Author	ID	Sex*	BMI [kg/m²]	Subtype	Resistance-associated mutations at baseline		CAB [ng/mL]	RPV [ng/mL]	HIV-1 RNA [copies/mL]
ATLAS-2 M [1, 12]	**1**	F	≥ 30	C	**RPV**:	**Major NNRTI RAM**	**1**,**100**	**74.0**	267
					**INI**:	n.r.			
	2	F	≥ 30	complex^&^	**RPV**:	n.r.	**650**	**14.2**	141,132
					**INI**:	n.r.			
	**3**	F	≥ 30	complex^&^	**RPV**:	**Major NNRTI RAM**	1,350	**34.8**	938
					**INI**:	Major INI RAM			
	4	M	< 30	A1	**RPV**:	n.r.	**921**	**34.2**	737,830
					**INI**:	n.r.			
	**5**	F	≥ 30	C	**RPV**:	**Major NNRTI RAM**	1,570	108	16,205
					**INI**:	n.r.			
	**6**	F	< 30	B	**RPV**:	**Major NNRTI RAM**	1,820	**44.3**	211,639
					**INI**:	n.r.			
	**7**	M	≥ 30	A1	**RPV**:	**Major NNRTI RAM**	1,450	**62.6**	5,687
					**INI**:	n.r.			
	8	M	< 30	A	**RPV**:	n.r.	1,440	78.5	296
					**INI**:	n.r.			
	9	M	< 30		**RPV**:	**Y181C**, K103N	**1**,**070**	118	n.r.
					**INI**:	None			
	**10**	M	< 30	B	**RPV**:	**E138A**,** M230M/L**	**837**	**36.0**	24,221
					**INI**:	Q148R			
	**11**	M	< 30	**A6**	**RPV**:	**E138A**,** Y181Y/C**	2,430	**57.0**	59,467
					**INI**:	Q148R			
	**12**	M	< 30	A1	**RPV**:	**E138K**	1,870	**48.5**	1,038†
					**INI**:	None			
Thoueille [6]	13	M	24.0	n.r.	**RPV**:	n.r.	**1**,**151**	**52**	17,000
					**INI**:	n.r.			
	14	M	24.0	n.r.	**RPV**:	n.r.	**704**	**55**	549
					**INI**:	n.r.			
	15	F	31.0	n.r.	**RPV**:	n.r.	**239**	**4**	62,000
					**INI**:	n.r.			
van Welzen [13]	16	M^#^	29.0	B & D (recombination)	**RPV**:	None	2,500	**5**	15,000
					**INI**:	n.r.			
	17	M	28.0	B	**RPV**:	179D	**420**	**33**	8,300,000
					**INI**:	n.r.			
	18	M	31.5	B	**RPV**:	None	**890**	**38**	9,400
					**INI**:	n.r.			
	19	M	27.0	B	**RPV**:	None	**260**	**20**	610,000
					**INI**:	n.r.			
	20	F	45.5	B	**RPV**:	None	**1**,**200**	690	620
					**INI**:	n.r.			
Bailón [14]	21	M	25.3	B	**RPV**:	n.r.	**330**	78	113,046
					**INI**:	n.r.			
Rubenstein [15]	22	M	29.4	n.r.	**RPV**:	n.r.	**701.3**	**27.7**	2,870
					**INI**:	n.r.			
Seang [16]	23	M	23.0	CRF02	**RPV**:	None	2,403	**44**	1,500
					**INI**:	None			
	24	M	25.0	n.r.	**RPV**:	None	5,188	**41**	327
					**INI**:	None			
Ehret [17]	25	n.r.	n.r.	**A6**	**RPV**:	n.r.	1,910	91	9,000
					**INI**:	n.r.			
	26	n.r.	n.r.	B	**RPV**:	n.r.	2,240	**48**	360
					**INI**:	n.r.			
Mazzitelli [18]	27	F	32	B	**RPV**:	None	3,180	152	358
					**INI**:	n.r.			
Gerstenberg [19]	28	M	19.8	B	**RPV**:	None	**778**	**51**	667
					**INI**:	None			

For Subtypes and rilpivirine-resistance associated mutations, red text color indicates the presence of a risk factor as identified in the previously published multivariate risk-factor analysis; ID numbers in red font indicate people with at least one of them being present at baseline. For cabotegravir and rilpivirine drug concentrations, red text color indicates a concentration below the data-derived threshold of 76 µg/L and 1,240 µg/L at the time of virologic failure, respectively

BMI: Body-mass-index; CAB: cabotegravir; M: male; F: female; n.r.: not reported; RPV: rilpivirine;

^&^: 3 or more subtypes present; *: Sex at birth; ^#^: transgender person; †: non-protocol-defined virologic failure

## Data Availability

Raw data are not publicly available to preserve individuals’ privacy under the European General Data Protection Regulation.
